# All that glitters is not gold: trustworthy and ethical AI principles

**DOI:** 10.1007/s43681-022-00232-x

**Published:** 2022-11-16

**Authors:** Connor Rees, Berndt Müller

**Affiliations:** Department of Computer Science, Swansea University, Swansea, UK

**Keywords:** AI ethics, AI regulation, AI security, Policy, Design guidelines, Human–AI interaction

## Abstract

Ethics of technology systems have become an area of interest in academic research as well as international policy in recent years. Several organisation have consequently published principles of ethical artificial intelligence (AI) in line with this trend. The documents identify principles, values, and other abstract requirements for AI development and deployment. Critics raise concerns about whether these documents are in fact constructive, or if they are produced as a higher form of virtue signalling. A theme that is beginning to become apparent in the academic literature regarding these documents is the inherent lack of effective and practical methods and processes for producing ethical AI. This article attempts a critical analysis which draws upon ethical AI documents from a range of contexts including company, organisational, governmental, and academic perspectives. Both the theoretical and practical components of AI guidelines are explored and analysed, consequently bringing to light the necessity of introducing a measurable component to such documents for the purpose of ensuring a positive outcome of deploying AI systems based on ethical principles. We propose a minimal framework for stakeholders to develop AI in an ethical and human-centred manner.

## Introduction

The purpose of artificial intelligence (AI) ethics is both human-centred, multifaceted, and in many ways diverse. Depending on the document there are different target audiences with different motivations and end-users in mind. Prior to unpicking the narrative shared by these documents, it is worth understanding what is meant by AI ethics more broadly. Although there are numerous depictions of an AI ethics definition, the following example has been selected on the merit of effectively encapsulating the complex notion. [1]:

“AI ethics is a set of values, principles, and techniques that employ widely accepted standards of right and wrong to guide moral conduct in the development and use of AI technologies.”

AI has gradually gained impact in recent years within numerous environments ranging from recommender algorithms to assisting medical diagnoses. Although its definition, implementation, and application have evaded a consensus understanding and standard, its fundamental purpose of being used for the common good has long been established. Isaac Asimov is said to have created the first governing laws for autonomous technology (robots) [2]. In his fictional book series ‘I, Robot’ published in the 1950s, three key principles were introduced which are now commonly referred to as Asimov’s Laws of Robotics. An additional principle (Law 0) was added in one of his later publications [3]. The four laws are as follows.

- **Law 0:** A robot may not harm humanity, or, by inaction, allow humanity to come to harm
- **Law 1:** A robot may not injure a human being or, through inaction, allow a human being to come to harm
- **Law 2:** A robot must obey the orders given it by human beings except where such orders would conflict with the First Law
- **Law 3**: A robot must protect its own existence as long as such protection does not conflict with the First or Second Laws

Despite originally being developed for a fictional environment, Asimov’s principles can be seen – to an extent – in a variety of modern-day publications. In seeking to refine and regulate Law 0, companies, organisations and governments are producing ethical standards, guidelines, and principles. In surveying these documents it becomes clear that they typically establish between 3 and 10 ethical principles, commonly referring to broad notions such as fairness, privacy, and autonomy. The necessity of ethics in the form of guidelines and principles has to be deemed paramount when dealing with technology such as AI as a form of check and balance to such a potentially powerful tool. However, most of these documents have shown a distinct lack of practical measures to assist in the adherence to the principles in systems design, as well as the monitoring and regulation of such systems.

Research in 2019 identified 84 documents on ethical principles or guidelines for AI [4], work specifically analysing AI guidelines has historically been less thoroughly and extensively explored. These publication form a new trend as is showcased by the majority of these publications being published after 2016 [4]. Existing literature on this topic includes:

- Comprehensive literature reviews to understand such documents [5]
- Identifying tensions between the company’s principles and guidelines [6]
- Potential improvements [7]
- The societal impacts of its application [8]
- Principles in conflict [9] among other avenues of exploration

This article complements the existing body of literature by identifying the absence of reference to practical applications of AI ethics principles and providing some recommendations based on these findings.

With existing literature finding consistencies between documents [4] on a large scale, this article takes an approach that considers the small scale. To gain a broad perspective from a limited sample size, four documents will be referred to in this analysis. These documents will provide a range of perspectives including a company (Google [10]), governmental (United Kingdom government [11]), organisation (European Union [12]) and academic (IEEE [13]). The justification for the broad range of document sources is to be able to identify the differences in priorities, the similarities in regulation, and potentially a solution that may be relevant to each entity regardless of their scope, scale, and demographic.

To enable a greater level of analysis and due to instances in the existing literature having compared larger number of documents, this article will focus on a select number of papers. Therefore, this article identifies four examples of AI guidelines as to most fairly and accurately represent their entity class. Thus, the critical analysis will begin with an understanding ethical principles, followed by a purpose and audience section expanding on the four previously identified documents, a subsequent section containing a comparison of common requirements and principles, a discussion chapter and finally a conclusion and future work section. The contents of which will address fundamental criticisms, and when combined suggest the need for trustworthy and ethical AI documents to include an application chapter or a separate accompanying monitoring and application document.

## Understanding ethical principles

Research regarding AI ethical principles is an evolving topic with increasing researcher attention. The critical analysis in this article considers these principles with regards to if/how these high-interest principles are set to be applied. It is first necessary to showcase an understanding of this topic, what research is being done, and what current views suggest about such guidelines and frameworks.

As AI is both practically and theoretically applied to an increasing number and range of environments and settings, its regulation becomes an increasingly pressing matter. As a result, the scale of the number of documents is ever-growing, with a study in September of 2019 identifying a total of 84 ‘AI ethics’ documents across the world that matched their inclusion criteria [4]. Accounting for a rate of growth, this suggests that there are roughly 100 documents currently in circulation. The utility of this increase in quantity - despite what one may assume - can be raised into question. This concern due to many of these documents referring nuanced terms and topics. This ultimately raises into question the utility of such documents and to what extent documents such as these are a higher form of virtue signalling or even dangerous. [7].

This ultimately calls into question the intention of the bodies that produce documents on AI ethics. Some academics have gone as far as to theorise that such documents effectively delay the bodies that produce them from regulating AI and instead shine the limelight on abstract problems and technical solutions [14]. Although this may be deemed both a stark and a drastic perception of AI ethics documents, it is not beyond reason. In a paper considering the ethical nature of AI ethics documents Hagendorff found policies not just by smaller bodies but as large in scale as Google to have vague and superficial principles set to provide a guideline and a framework, but in actuality suggests and provides little in the way of practical resources to those creating ethical AI systems [15].

The recent surge in AI ethical principle documents may seem to be a step in the right direction. However, things might not be what they appear. These documents largely refer to the same nuanced concepts that are produced without any practical implications. In engaging with these documents there is an innate presumption that the proposed principles will directly translate into practice, however, this is very rarely the case [16]. An alternative approach could be to have an understanding of AI-specific methods and practices to set the mold, and implement realistic and achievable goals around them.

An implemented example of this can be found in the medical sector. Overarching virtuous principles are uncommon in clinical decision making. In contrast, institutional policies and principled concerns are used to inform and regulate such processes [17]. Although these are both inherently different fields, the similarities regarding the ethical principles have been explored in some detail, as set out in a recent review which found a close link between the four classic overarching principles found in medical ethics and the principles found in many of the AI ethics documents [18]. Thus, in seeking to conceptualise the landscape of AI ethical principles, there is an understanding that the existing landscape of work is subject to a range of criticisms. With this in mind, the following chapters will consider each of the four different AI ethics guidelines. In doing so, the overarching principles and requirements will be considered in addition to the measures provided to apply and enforce said principles, that is, if these sections are present.

## Purpose and audience

To position the previously identified four AI ethics documents studied in this article [10, 12, 19, 20], it is necessary to explore the each source’s intended purpose and audience. This is necessary due to ethical AI principles having not yet reach a consensus definition and scope. In doing so, this will frame the principles within the specific context that each document intended.

***Google***

In mid 2018, Google released a post on their blog platform about their own ethical AI objectives in a document title ’Artificial Intelligence at Google: Our Principles’[10]. Google sets out 7 fundamental self-established principles as a foundation for the company to use alongside future developments of AI. As self-proclaimed leaders in AI, the company’s CEO voices a sense of responsibility in developing ethical AI. The post also establishes an understanding of criticisms of such principles through relaying their awareness of critiques around the theoretical concepts seen in other sources of such principles. However, they claim that the standards actively govern their in-house research and product developments and will also have a level of impact on business decisions.

***UK Government***

In the document produced by the UK Government [19], AI ethics are referred to as the ethical building blocks needed for the responsible delivery of an AI project. The foundations of these principles are built on the understanding that AI ethics emerged from the need to address the harms AI systems can cause, namely applications that invade users’ privacy [19]. The principles are aimed at anyone involved in the design, production, and deployment of an AI project including but not limited to data scientists, data engineers, domain experts, delivery managers, and departmental leads. The document sets out four principles that were developed in correspondence with the Alan Turing Institute’s public policy program [20] who themselves have their own AI ethic principles [1]. These principles are set to be partnered with other documents as referenced above in addition to the UK’s data ethics framework [11] which supplements these principles with specific actions. The government implies the difficulties in feasibly protecting privacy through AI principles developing several documents as opposed to one condensed principle document.

***EU***

The document produced by the EU is a far more diverse and multifaceted approach to ethical AI than what is commonly seen in such documents [12]. To achieve trustworthy AI throughout the entire life-cycle of a system is must be lawful, ethical, and robust. Thus, the ethical principles found in this document are supplementary as opposed to being the fundamental quality as they make up a third of the total requirements to achieve trustworthy AI. The principles are primarily aimed at the developers, deployers, and end-users of the system, in addition to the wider society. However, each stakeholder has a specific role. Developers are expected to implement and apply these principles, deployers are required to ensure that the systems uphold principles and inform end-users about the principles of which there are three: lawful, ethical, and robust. These principles are subsequently expanded into a further seven ethical requirements, similar to the UK government approach.

***IEEE***

The IEEE’s 2018 Ethically Aligned AI [13] (now available in the first edition, [21]) is simultaneously clear and ambitious in stating the aims and aspirations for what is referred to as their ’General Principles’. These principles apply to autonomous and intelligent systems, including physical robots (e.g., driverless cars) and software systems (e.g., algorithmic chatbots). However, the principles are not aimed at one specific group or stakeholder. The purpose of the principles is to underpin future norms and standards regarding ethical governance. Therefore, the target audience is those developing their own standards, those looking to regulate standards, and to those researching the epitome of ethical standards within this context. Thereby, the IEEE showcases a document that not only communicates high-level guiding principles but also some micro-level information with accompanying contextual, cultural, and pragmatic solutions regarding implementation.

## Comparison of common requirements and principles

Upon scrutinising each of the four documents, it becomes apparent that a two-tiered structure was adopted in each of their developments. The first of these two tiers are the AI ethics principles, the second tier identifies actionable requirements in line with the first tier. As a result, the formatting of this section will be partitioned into two sections, (a) principles and (b) requirements. In doing so, these sections will identify common characteristics in the strands of these documents. This comparison process will not address all of the principles available in each document, but focus on the primary groupings of common themes using a unified definition. These themes will be presented through tables using the key displayed in Table 1.

**Table 1:** Classification symbols and their definitions

Symbol	Definition
✓	Specific reference has been made
✓✓	Specific reference has been made in more than one principle
$\mathbf {\square }$	Non-specific reference has been made
✗	Not present in the document

### Principles

The principles element of the two-tiered breakdown of these documents highlighted five key common groupings. These concepts were shared by at least two of the documents to qualify to make the list, as can be found in Table 2. The five common principles found in these three documents consist of: ‘Robustness’, ‘Lawfulness’, ‘Human-centredness’, ‘Fairness’, and ‘Accountability’. The selected definitions of these terms were formed from combinations of the each of the original definitions and descriptions found in each of these individual documents.

**Table 2:** Common Principles and their presence in organisational ethical documents

Principle	Description	Google	UK	EU	IEEE
Robustness	Regarding technical perspective and the social environment	✓	$\mathbf {\square }$	✓	✓
Lawfulness	Respecting of applicable laws and regulations	✓	✗	✓	✓
Human-Centredness	Accommodating of the need of the individuals whom it impacts with social benefit in mind	✓	$\mathbf {\square }$	$\mathbf {\square }$	✓✓
Fairness	In all steps of the process including data, design, outcome, and implementation	✓	✓	✗	✗
Accountability	Requirement of full accountability	✓	✓	✗	✓

The robustness theme summarises principles within the documents which identify the requirement for sufficiently developed systems. The concept behind this notion presumes that by making a vigorously developed and tested system will limit the negative fallout of its implementation. However, despite its presence in Google and the EU’s (and to some degree within the UK’s) document, to require robust AI is akin to telling a human to be good. It depends on the understanding and interpretation of the requirement within a context. Arguably, the question of whether a human is good cannot be answered without specific metrics as a mode of monitoring this criterion.

Including the law in any facet within an ethical system is an interesting position to take due to the more common segregated position that law and ethics take as concepts. The UK document follows a more traditional route through keeping these two topics separate in its principles by choosing to not mention of the law in its publication. In contrast, Google’s and the EU’s principles make specific reference to abiding by the law, simply conveying that a system cannot be ethical or unlawful to any degree. The IEEE takes this one step further, with lawfulness being incorporated in two of the ethical principles. Through incorporating this principle there is a question left unanswered which poses whether the legality requirement is automatically met unless a law enforcement agency states otherwise, or is this a requirement that must have its own provisions set out by the source of the system? This is certainly left unclear and unanswered.

A human-centered approach is an imperative requirement to incorporate within ethical processes [1]. Thus, the first principle in Google’s document is to ‘be socially beneficial’, when pairing this with frequent reference to stakeholders it becomes clear that to a degree having a human-centered approach is to have an ethical approach. Although not identified there are many avenues to measure this principle; however, such measures are not without limitation. In that, AI can be re-purposed in a way that reverts this principle. Thus, the task of meeting this criterion and the many avenues it explores becomes a far more convoluted task open to much interpretation.

Fairness is the first of four AI ethics principles in the UK document and although not present in the EU document, reference is made to avoiding the creation or reinforcement unfair bias is present. There are many avenues of fairness i.e., data, outcome, implementation etc.; however, the ingrained subjectivity of this term cannot be understated. Thus, where these principles provide examples of where fairness can be applied, it is of very little benefit when establishing how the principle can be adhered to and regulated.

The very essence of accountability ties in with the issue surrounding lawfulness. Google identifies the need for lawfulness whereas the UK document does not. In the context of Google’s principles, to be lawful and accountable refers to the same principle, in that being lawful makes you accountable to a law enforcement agency. With accountability not being present in the EU’s principles, it may be assumed that the presence of lawfulness may justify the omission of explicitly mentioning accountability. On the other hand, the UK document does not identify lawfulness explicitly within its principles, relaying this aspect to existing legislation. Thus, identifying the need for accountability may be deemed essential despite its nondescript nature. Identifying to whom a system is accountable and the remit and scope of such a notion consequently becomes the primary area of interest.

Based on these groupings, it is the interpretation of this article that the findings are in line with those drawn from the understanding of ethical principles sections. Through analysing the five themes: robustness, lawfulness, fairness, accountability, and human-centeredness; the nuanced and non-specific nature of these principles becomes inherent. One of the many limitations of the principles is that despite researchers’ best efforts to segregate each principle, there are clear cases of cross-pollination from one principle to another. For example, to be robust may include being accountable; the same way that being lawful and fair may be deemed interchangeable in a given context. Although the implementable parameters that embody these themes will be analysed in the following subsection, the use and benefit of the inclusion of these principles beyond virtue signalling is raised into question.

### Requirements

Principles need to be underpinned by guidelines and requirements to ensure their processes are actionable. This holds equally for AI ethics principles. This section analyses the requirements that follow the principles and provide more specific demands that expand beyond simply adhering to the ethos provided by the principles. Google’s requirements are structured under the seventh principle, while the UK has a separate document containing the principles in a data ethics framework [22] and both the EU and the IEEE embed the requirements into their principles document. Akin to the principle in the previous section, requirements are grouped together, summarised, and analysed. The intended purpose of these requirements is to expand on the principles. Hence, this section queries to what extent this is the case or whether they are equally limited as the principles themselves in isolation. An overview of the most important requirements and their coverage is shown in Table 3.

**Table 3:** Common Principles and their presence in organisational ethical documents

Requirements	Description	Google	UK	EU	IEEE
Safety & Impact	Regarding technical perspective and the social environment	✓✓	✓✓	✓✓	✓✓
Diversity	Respecting of applicable laws and regulations	✓	✓	✓	✗
Accountability	Accommodating of the need of the individuals whom it impacts with social benefit in mind	✗	✓	✓	✓✓
Legality	In all steps of the process including data, design, outcome, and implementation	✗	✓	✓	✓✓

The requirements which fall under the safety and impact category are the most prominent in each of the documents. Multiple principles developed this sentiment of minimising unintentional harm. Due to the multifaceted nature of AI, eliminating harm in its entirety may be an impossible task. The ethical requirements make a clear effort to minimise unintentional harm, the difficulty in the sense is that you cannot minimise harms that you are not aware of or cannot see. However, by producing a public report that details these efforts, such efforts may be better sold as working documents as opposed to something ‘achieved’ *per se*.

Diversity as an ethical requirement in terms of AI development is not a complex process to engage in. However, making diversity a meaningful tool for avoiding discrimination and achieving fairness is far more convoluted. Notably, developing unbiased data is an exceptionally difficult – if not impossible – task. Furthermore, to exclude discrimination may be to the benefit of fairness but to the detriment of quality and accuracy. Certainly, a compromise can be sought in this context, however, there is little to no evidence in these requirements of drawing a line and being open to feedback and constructive criticism. All documents fall short of concrete steps to facilitate diversity as a means for achieving better or more ethically aligned AI.

Both accountability and legality are covered as categories for ethical requirements along with their existence as ethical principles. The primary concern for these two aspects previously was the specificity and measurability. This concern persists in this setting where requiring their existence without guiding resources is not vastly different to asking someone to create an AI system without prior training. Although one can try, the results are certain to be sub-optimal, particularly in the case of accountability. The nature of which is very unclear when it comes to AI, as a result including accountability without specificity provides little to no practical benefit. In particular, the question of machine accountability is not covered at all.

At every step of the process of designing ethically aligned AI, the theme of nuance versus non-specificity becomes increasingly significant. It can be argued that something is better than nothing, and so these documents provide a basis to discuss the parameters and scope of ethical regulation and requirements. However, until there is an example of a document that states specific requirements, concrete achievements and actions this discussion is limited to theory as opposed to practice. Undoubtedly, the current activities around standardisation and regulation of aspects of AI, as well as AI in specific contexts, such as healthcare, will provide more detailed guidelines for developers.

## Comparison of application and measurement

Having identified themes and trends in the representative AI ethics documents’ understandings of key concepts alongside their specific implementability, this section identifies the applicability of the ethical principles and how each body applies each of the principles. Furthermore, this section incorporates the measures required to monitor their application. Digging beneath the surface, it becomes clear that these characteristics are not a covered in any of these documents.

The main criticism of Google’s AI principles is the absence of clarity regarding its application. It is worth re-iterating that this is not a guide for others (although it talks about working with others), but rather a transparency blog to share what Google considers when developing ‘ethical AI’.

In a separate blog, Google discusses applying its aforementioned principles [23]. Four key methods of application are introduced: internal education, tools and research, review processes, and working with external stakeholders. These points are also developed in a blog where Google discusses its AI principles six months after their application [24]. Although these blogs highlight clear constructive steps to developing ethical AI, such as removing more bias from Google Translate and developing ‘Cloud Hub AI’ to share new machine learning training models. The blogs and Google’s document fail to identify specific ways in which all of these are linked. This may not seem essential, however, with no specific reference to the seven principles but to ethical AI in general, it raises into question what the purpose of these principles was in the first place.

The four principles provided by the UK government are set out to be building blocks for ethical AI. As such, they are each broken down into several practical ‘standards’ for an AI developer to achieve. Although some are more nuanced than others, the principles do not sell themselves as more than they are; a starting point. The references to further material made throughout, pointing developers in the right direction; ultimately calls into question why the principles were established at all. Alternatively, the UK governments could have developed a hub for ethical AI with links to specific documents regarding specific concerns.

In terms of application, the document proposes an overarching ‘process-based governance framework’ following the same building blocks theme. Whereby it suggests for each governance action having a designated member of staff, targeted considerations, time frames, clear and well-defined protocols. Although not detailed and adequately fruitful, the UK Government’s ethical AI document signposts developers to good practice and a robust foundation. This again brings into question the purpose of the four principles.

As previously identified, the principles set out by the EU are aimed at the developers, deployers, and end-users of an AI system. In critically analysing the key concepts referred to in these principles it becomes clear the scope is too broad to achieve measurable principles. With some principles being more relevant and accessible to one demographic, this leaves the others left with questions. Thus, suggesting that three different documents could have been developed to best suit each demographic and the same goes for its application.

The implementation and application will naturally vary based on each of the three demographics. However, these rights, principles, and values are identified as one of four key steps to realise ethical AI. This followed by setting requirements for ethical AI, technical and non-technical methods to implement the requirements, and finally, a continuous evaluation and justification process made up of use, analysis, re-design, and development. Although the EU identifies that these principles have a place in the foundations of ethical AI, ultimately there is incoherency and a lack of clarity in who each point is targeted at, how specifically they are to be upheld and what methods will be used to monitor them beyond being voluntarily conducted in-house.

The document produced by the IEEE, as previously noted, clearly stated its purpose as providing the standard by which other similar documents can be gauged and compared to. Certainly, this differs from the other examples in that there is no clear route to evidencing or disproving whether or not this can be achieved. Having made that clear, the inherent thorough nature of the principles sets it apart from the rest. Through framing each principle in response to an issue, providing background information, recommendations, and further resources. Through the representative comparison conducted by this article, it may be deemed that the IEEE achieves its aims and purpose. However, things cannot be said with absolute certainty without a systematic comparison comparing each of these ethical principle documents.

In regards to the application of the principles presented in this document, a clear effort has been made to circumvent specificity. The application and implementation of the ethical principles is thorough but not specific. In that, through the candidate recommendation element present in the description of each principle, such matters are addressed and identified. However, the approach this document has opted for applies the principles to specific examples in contrast to providing a broad framework as can commonly be found in other examples. Conversely, this does offer a level of specificity that is not present in other documents. Therefore, this simply shows that there can be no one size fits all within this context.

The application and measurement of the principles found in these documents alongside their accompanying requirements, fail to show an example of an organisation taking ethical requirements seriously. Organisations signpost and point in a direction without providing hard requirements, processes, or frameworks. The absence of fixed requirements and inclusion of broad notions reinforces the accusation that in many cases these documents provide little more than an opportunity for large organisations to virtue signal whilst failing to prevent potential harms.

## Impacts on current policy

This section summarises some recent UK and global developments that can be seen as a direct consequence of the discussions on AI Ethics Principles. Although this section cannot claim to be exhaustive, but exhibits the accepted need for guidance that will inevitably lead to standardisation and regulation in the near future.

### United Kingdom

#### ICO

Privacy as part of the ethical foundation for technological solutions and also as part of the Cyber-security considerations for responsible innovation, has been addressed by the Information Commissioner’s Office (ICO), the UK’s public body reporting directly to Parliament and sponsored by the Department for Digital, Culture, Media and Sport. The main focus has been on designing in aspects of data protection and privacy from the conceptual stages of technology development. This can be seen in the publication of various checklists, such as “Data protection by design and default” [25] and “Data protection impact assessments”[26].

Privacy by design was first introduced by the Information & Privacy Commissioner of Ontario in the 1990s and found a resurgence in the 2010s, as evidenced by a communication [27] in 2013 highlighting 7 principles of responsible design, naming 9 key application areas, including surveillance systems, biometrics, smart meters, mobile devices & communications, Near Field Communications (NFC), sensors and geolocation, healthcare, and big data & data analytics.

This is also emphasised by the European Union Agency for Cybersecurity (ENISA) in various publications dating back to 20125 and including postquantum cryptography as a major challenge in February 2021 [28].

#### UK AI strategy

The UK as of 2021 published its new AI Strategy. The strategy is based on the work of the AI Council [29], an independent expert committee advising the UK Government. The AI Council was founded in 2018, following publication of report [30] of the House of Lords Select Committee on AI, to reflect all aspects of AI in the public and private sectors, as well as in academia. In January 2021, the AI Council published an AI Roadmap [31], highlighting four areas of political support for AI in the UK:

1. Research, Development and Innovation
2. Skills and Diversity
3. Data Infrastructure and Public Trust
4. National Cross-Sector Adoption

In all but the last item, some aspects of ethical design are reflected, ranging from interdisciplinary, over democratising AI, avoiding bias and promoting fairness, to transparency and explainability.

### Beyond the UK

#### Europe

*Ethics Guidelines for Trustworthy AI* were published by the High-Level Expert Group on AI of the European Commission in April 2019 [12], with the separate publication of a *Trustworthy AI Assessment List* [32]. The former being one of the sources of ethical AI principles, however the implication of this document and the subsequent trustworthy AI assessment list may be more direct than other examples of such documents. These examples can be seen as the initial trigger for the Proposal for a *Regulation of the European Parliament and of the Council Laying Down Harmonised Rules on Artificial Intelligence (Artificial Intelligence Act) and Amending Certain Union Legislative Acts* published on 21 April 2021[33].

#### IEEE P7000 series

The first instalment of IEEE’s P7000 series [34] marked the first ethical standard of ethical issues in system design [35]. There are a total of 15 standards in this series with the most recent amendment to IEEE 7002-2022 on the Data Privacy Process. The series aims to provide project stakeholders with a methodological process to managed ethical concerns throughout a projects’ life-cycle. This methodology proposed in the series identifies the analysis of ‘values’ or ethics principles to inform on and refine ethical system requirements.

#### World economic forum

The World Economic Forum have designed the Chatbots RESET Framework for conversational AI [36]. The basis of this framework builds on both the World Health Organization and the Centers for Disease Control recent adoption of chatbots as a result of the global pandemic. To cater for the increase in demand for coronavirus information symptom checking. In addition to the global reach of these organisations, national governments and healthcare providers began to implement the use of chatbots to provide relief on resources. As is the case in a number of contexts, the fallout of technical solutions as a result of the pandemic is likely to have a lasting effect after its passing; the adoption of chatbots in a healthcare context is theorised to be continued after the pandemic has passed [36].

In all but the last item, some aspects of ethical design are reflected, ranging from interdisciplinary, over democratising AI, avoiding bias and promoting fairness, to transparency and explainability

The RESET framework offers 10 chatbot principles. In this collection the common principles are that of human agency and oversight, fairness, accountability and safety. Ethical design is explicitly mentioned in this framework. It is exemplified and accompanied by operational actions than can be followed to obtain said principles, thereby supplying more practical guidance than the high-level document in the main comparison of this article.

#### UNESCO

In June 2021, the United Nations Educational, Scientific and Cultural Organization (UNESCO) published the second revision of a draft for a *Recommendation on the Ethics of Artificial Intelligence* [37]. This was ratified in November 2021 by the General Conference of the UNESCO [38]. The importance of this recommendation becomes clear from the following quote by the UNESCO Director-General Audrey Azoulay.

   “The world needs rules for artificial intelligence to benefit humanity. The Recommendation on the ethics of AI is a major answer. It sets the first global normative framework while giving States the responsibility to apply it at their level. UNESCO will support its 193 Member States in its implementation and ask them to report regularly on their progress and practices.”

Key points of the recommendation are:

- Data protection;
- Banning social scoring and mass surveillance;
- Helping to monitor and evaluate;
- Protecting the environment.

*Ethical Impact Assessment* is at the heart of the documentation and manifests itself in the goal of assessing the impact AI systems have on individuals, on society, and on the environment. The UNESCO envisages providing a tool to support governments and other organisations ensure that systems follow ethical principles. It is also recommended that member states should appoint an independent *AI Ethics Officer* or implement other mechanisms to ensure meaningful auditing is carried out.

### Translation of principles into standards frameworks, government policy, and legislation

We compare the numbers of occurrences of keywords related to responsible and ethically aligned design in the principles documents and compare this to a very recent publication on forthcoming AI regulation by the European Commission and the UK AI roadmap. Key concepts in this study included:

- privacy,
- security,
- safety,
- protection,
- human-centeredness,
- trust,
- transparency,
- ethics,
- bias,
- diversity,
- fairness,
- liability,
- accountability,
- accuracy.

In [39] we introduced technology design following the ${\textbf {Re}}^3$ principles of *reliability*, *responsibility*, and *resilience*. It is worth considering grouping the keywords we used to characterise the AI principles documents according to this approach. Many of these aspects are related to one another, but it has proven useful to consider the following groupings.

- *Reliability* of AI-driven systems can be achieved by facilitating

- use of **formal methods** leading to provably correct systems where this is feasible;
- extensive and **systematic testing** leading to a significantly lower rate of functional errors;
- **diversity in design** leading to fewer unexpected behaviours.

- *Responsible* design of AI components should be based on

- legal and ethical **compliance by design**;
- **explainability** to enable understanding of decision-making processes;
- identification of a natural or legal person who is **accountable** for the system.

- *Resilient* systems require

- **safe** and **secure** design of AI-driven components;
- **robust** systems design and **recovery** strategies.

All of the above contribute to trust in data-driven and AI-based systems. The groupings should not be seen as a partition. In fact, there are numerous inter-dependencies, e.g., explainability can be achieved by use of formal methods such as argumentation systems; formal methods can be instrumental for the safety of systems; diversity can help ensure legal and ethical compliance, and fairness)

**Fig. 1:**
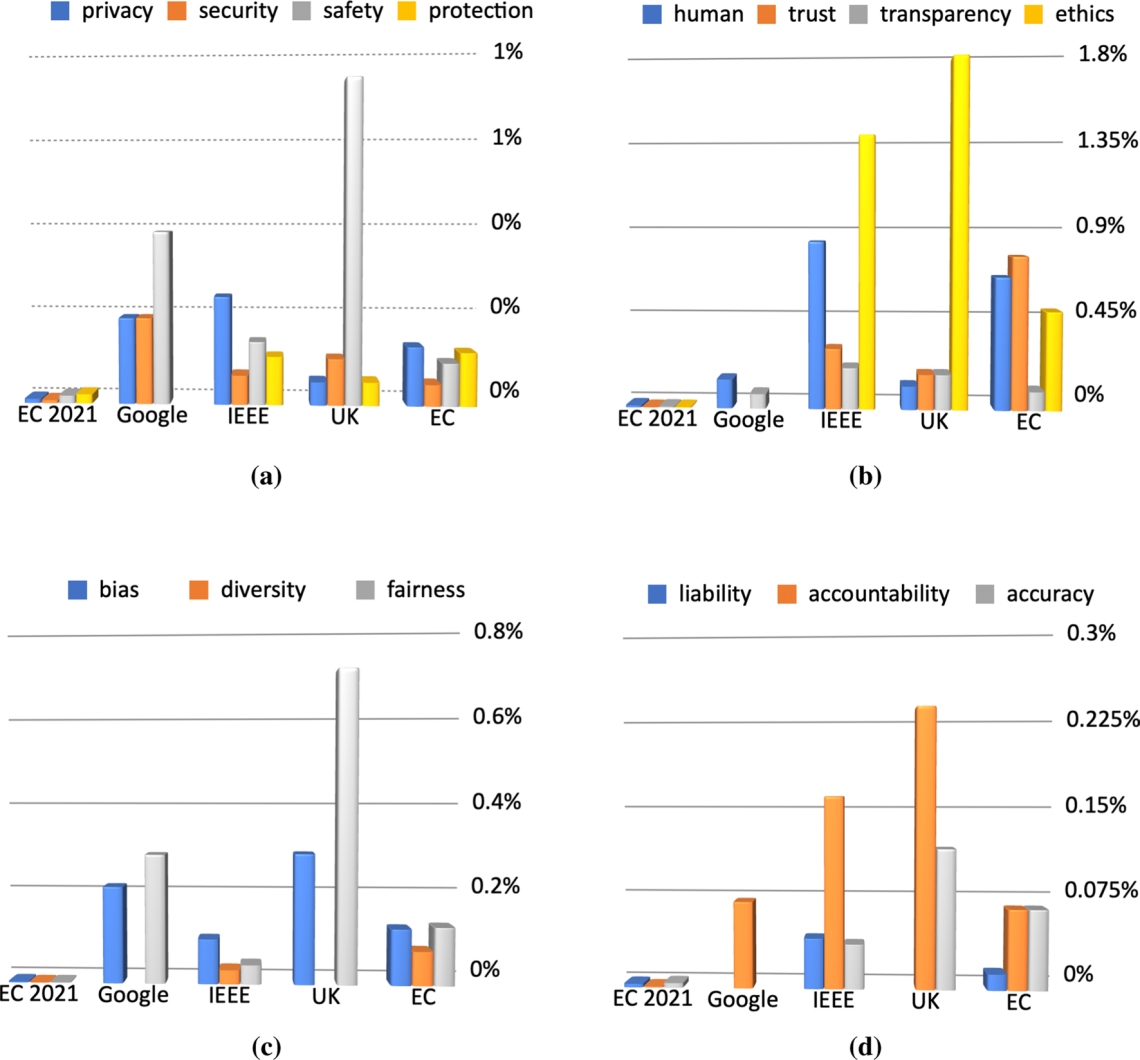
Frequency of keywords as a proportion of the document length for the sample AI-principles documents: EC2001 [33], Google [10], IEEE [13], UK [11], EC [12]

Figure 1 illustrates the prominence of these keywords in the various documents. This highlights some interesting facts relating to priorities of the organisations behind the documents and the expected translation from legally non-binding principles to actual policies and regulation. We discuss some of these trends below.

The early European Commission document [12] immediately stands out with a much lower proportion of the keyword ‘transparency’ compared to ‘ethics’ and ‘trust’ in Fig. 1b. The reason for this is its intention to start a conversation about ethical aspects of implemented AI systems rather than means to achieve these. The EC 2021 document is to be regarded as an outlier, since it is a proposal for an EU regulation. As such, it has to be a lot wordier and align the human-centred ethical aspects with the interests of economy, ecology, and general legislation. It is clear, however, that despite the low frequency of the keywords, all aspects are present and treated with a similar level of importance, assuming this correlates with the keyword frequencies (Fig. 1a–d).

It is worth pointing out that the UK document and the Google document highlight the importance of fairness and link this to bias, but both show weaknesses in making the connection to a diverse AI development team and diversity in the evaluation environment (see Fig. 1c. Given the UK’s history of health and safety regulations, it is not surprising that the UK document emphasises the safety of AI-driven processes (Fig. 1a). The UK, IEEE, and EC documents also puts significant weight on the accountability aspect, combined with the question of accuracy and liability (Fig. 1d). This is less developed in the company document, hence justifying a call for regulation of AI.

## Findings

The intended target audiences for each of the AI ethical principles documents illustrate slight variation, whereby some are applied broadly to AI and others to more specifically to robots and/or AI, or even more broadly to automated machines. At their core, these can be viewed as strictly different entities that have some similarities. The more easily understood distinction between AI and robotics can be simplified as AI operating in the digital space and robotics in the physical space. These two broad examples of technology in the context of ethical considerations form a symbiotic relationship [40]. Certainly if there is a difference and there is scope for these two notions to be considered separately in future, more specific requirements for designing-in ethics into such systems is an approach to consider (ethics in design underpinning a culture of ethics by design). However, until a broader umbrella of established ethical conduct can be achieved, it is arguable that there is little benefit in separating these two concepts.

As previously identified, despite the recent surge in AI ethical principle documents appearing to be a step in the right direction, all that glitters is not gold. The criticisms regarding nuance and non-specificity constitute a pair of themes that are consistent throughout all of the four documents. This is a key finding, apparent in all of the four different sources, despite each being designed with a different intended purpose or audience: Google’s document seeks to inform the public of self-imposed standards and achieve trust in their products; the UK Government’s principles as a supplementary document to their ethical guidance document aiming to achieve a broad political consensus on terminology and ambitions around AI ethics; the European Union’s document aiming at stakeholders of any facet of an AI systems’ life cycle (developers, deployers, end-users, and wider society) and emphasising the global nature of the problem; and finally IEEE’s principles apply to all types of autonomous and intelligent systems and providing several example domains with the most nuanced discussion of all of these documents.

Each of the documents identifies principles followed by several guidelines or requirements to accommodate the principles. However, the inherent lack of concise detail presented itself throughout each of the documents. There was no identification of who would be responsible for enforcing the suggested measures, which ones should be mandatory and which could remain optional, how they could be achieved, or even how they could be measured. It is only apparent that they existed and that an AI system, to be ethical should abide by them...an inherently problematic concept. In some ways, the principles documents highlight problem areas of AI – which is valuable in its own right, but does not provide concrete steps to tackle these problems. As became clear throughout this critical analysis, many of the principles and the objectives which fell within these principles are simply immeasurable. There is currently no method of effective application of the principles beyond placing broad responsibilities on designers, developers, and users of AI systems.

The responsibilities which fall under the remit of these principles are more specific and in many ways more constructive. These requirements state specific criteria that state how AI should be developed or is developed in an organisation. This is evidently more than what is offered by the principles themselves, however, there are still limitations that need to be addressed for a regulatory framework. Some pertinent questions emerge from the problem of how to measure, gauge, record, and report these factors. Many of the principles offer no support on how to achieve the requirements they postulate. Thus, the development of a catalogue of measures that could be put in place for each of the ethical principles is necessary. The main obstacle of this is that there are many different contexts and many different AI solutions that would all have to be considered separately and in combination. The multitude of possible scenarios will make definitive answers or approaches impossible.

As a good chef will have developed a refined method of judgement when it comes to ingredients and preparation of their signature dishes, a sophisticated AI developer will have to acquire a sense of judgement concerning the ingredients of the AI-enabled solution they are intending to build. To ensure food safety standards, a system of traceability has been put into effect by some nations and organisations (such as the UK Food Standards Agency and supermarket chains). A similar system making the origin of data traceable needs to be implemented when dealing with AI. In doing so, raising the idea that similar to practices in the field of security, one may consider the option of creating voluntary transparency about the use of unbiased bodies of data that could offer a first step towards ethical certification. Beyond self-certification, it would be necessary to create authoritative bodies to check and provide such certification.

In the years following the surge of AI ethics principles being suggested and adopted beyond academia and into private, corporate, and governmental sectors, the realities of these ethical principles are that, regardless of their origin and initial impact, in the current climate of AI euphoria combined with fears about negative impacts of AI on humanity, we are seeing an unprecedented degree of impact on current policy on a global scale. Although measuring the impact and the degree of uptake is not realistically possible, it might not be necessary either. As long as these principles are widely recognised, adopted, and accounted for in a broad spectrum of contexts, the intended result of human-centred, ethical technology, and AI is at arm’s length. This can only be judged by transparency.

One of the findings of this article is that in each of the representative examples of principles developed by different types of organisations, successful implementation goes hand in hand with accountability. However, there is little to suggest any evidence to what extent such principles offer measurable utility. Therefore, although the proclamation of AI principles has no real negative impact, a lot of effort needs to be invested to enable us to determine if these principles should exist and – more specifically – what purpose they serve. This re-iterates one of the concerns raised in Chapter 4.1: Should we move away from releasing ever more sets of AI ethical principles and towards identifying steps that need to be put in place to ensure they are adhered to, monitored, and regulated.

The various sets of principles for AI discussed above define the cornerstones of a design, development, and deployment lifecycle based on reliability, responsibility, and resilience [41]. Technological advances in recent years have brought about methods that address the major issues in technology ethics and that should be incorporated into systems engineering frameworks. These techniques include, but are not limited to:

- Federated learning [42]
- $$\epsilon$$-differential privacy [43]
- Counterfactuals [44]
- Argumentation theory [45]
- Explainable AI frameworks based on game theoretic optimisation, e.g., Shapley values [46], and local surrogate methods, such as LIME [47].

All of the recent policy developments call for a combination of learning and reasoning in AI to assist in enabling the evaluation of current technology for its underlying ethical standards.

Most of the above considerations are based on AI systems, but should the legislation and regulation governing the ethical principles be limited to AI (when we still do not actually have a good definition of AI) or should this incorporate any form of ‘computation’? Powerful AI can exacerbate some of the issues concerning bias and unfairness making them systemic. AI is fast becoming ubiquitous and all reasonably complex computational systems are prone to suffer from some biases, often unintentionally introduced by the developers. This means, it is likely that the distinguishing factor of systems ‘using AI’ will soon become obsolete, just as nobody would point out the fact that they work in ‘electronic’ data processing, a popular term in the 1980s because it will be seen as a given that systems facilitate some form of AI. While automation without autonomy might not be considered as AI by some, the ethical considerations should affect all kinds of technology.

It is becoming increasingly clear that in addition to a set of ethical principles, there is a need of tried-and-tested industry best practices. Since these can not be expected to be all-encompassing, it would be desirable to create a repository of case studies, that go beyond anecdotal treatment of problems or successes, from which organisations can learn. Similar repositories have proven to be indispensable for making informed choices in the areas of cyber security (e.g., the Open Web Application Security Project – OWASP [48]) and privacy (e.g., the Chronology of Data Breaches [49]). Omissions in adequate ethical alignment can have similar consequences as neglected vulnerabilities in cyber physical systems. Presently, it is predominantly the reputation of organisations that is at stake (with the indirect consequence of loosing users due to loss of trust), but in the near future there will be more direct financial impacts due to the likely introduction of penalties for negligent design and deployment of AI systems and accountability/liability being formalised into legislation.

Following the discussion above, we would like to recommend a minimal set of requirements for ethics-by-design systems engineering. It is important to note that these requirements should scale proportionately to the size of the workforce and to the scale of the project:

1. **Embrace the diversity of diversity**: The emphasis should be the realisation that diversity does not only include the aspects of ethnicity, age, and gender but also socio-economic situation, geography, culture, religion, as well as skills, and methods. This is a fundamental requirement for any complex AI-based system to (a) avoid unconscious disadvantaging of subgroups, (b) explore suitability of solutions in different (cultural) contexts, and (c) exploit novel approaches an re-think existing processes.
2. **Future-proof systems** by considering likely introduction of legislation around accountability and ethical AI systems design. Organisations will benefit from demonstrable compliance with emerging industry standards (e.g., IEEE 7000 series [34]) in addition to compliance with local and global legislation.
3. **Re-assess ethical compliance regularly:** Proactively and voluntarily engage in and document periodic or continual checking of ethical (and legal) compliance of evolving, learning, and autonomous systems. View this as an M.O.T.^1^ for data-driven and AI-based systems [41].
4. **Summarise in laypersons’ terms** how the decision making process is supported by AI, how quality control is continually ensured, and where human oversight is employed, as well as what data is used and their provenance. This should demonstrate traceability and fairness where possible, and help enhance transparency, which – in turn – leads to increased user acceptance.

Addressing each of these four requirements in the development of ethical AI would thereby meet a minimum standard as set out by the Re$^{3}$ principles of reliability, responsibility and resilience. In an attempt to comply with current and future regulation (item 2 above), the steps taken should be documented. For diversity (item 1 above), this could be simply a performed by filling in a checklist of types of diversity (e.g., ethnicity, age, skills, etc.) and the level of engagement (e.g., diversity within the design or development team, diversity on a panel overseeing the development, retrospective ethical approval by a diverse panel within the organisation or by user surveys, etc.). Documentation of item 3 would require the specification of a strategy for re-assessing the deployed system, as well as a mitigation strategy addressing cases in which a re-assessment shows ethical misalignment. Essentially, the documentation should go hand-in-hand with a cyber-security risk assessment strategy. While neither security risk assessments nor ethical assessments are compulsory in all areas in which AI is employed, the benefits have been widely recognised by public and private organisations alike.

## Conclusion and future work

This article has sought to critically analyse organisational, company, governmental and academic ethical AI documents through comparing principles and requirements. In doing so, it has identified the understandings established in the academic literature to conceptualise the principles and their respective applications. Although it is not within the scope of this article to consider the criticisms regarding whether these documents are a virtue signalling display, several conclusions can still be drawn.

This article has shown the recent history of ethical guidelines for the development of AI and other technology. It can only provide a snapshot of what will become a dynamically evolving field of recommendations and regulations as more organisations and government commit to legally binding ethical principles. Similar to previous developments in cyber security, we have reached a maturity of technology and insights that demand a radical change in the way technology is created. A similar analysis on a broader scale with a larger corpus to reinforce the results and findings could be carried out. However, the pace of advancement will always mean that some new insight will be reserved for future investigation. This reflects the need for re-assessment in cyber-security reconnaissance and similarly the need for continuous monitoring and re-alignment of decision making processes in AI-based autonomous systems. Furthermore, an analysis of how well AI ethical principles align to AI frameworks produced by the same or other bodies will have to be scrutinised. Identifying and scoring how well the application of ethical AI frameworks corresponds with agreed ethical AI principles could be used to increase transparency, ideally leading to clear labelling of systems (similar to food labelling in the UK). Regulatory aspects and certification of compliance with ethical principles or frameworks could potentially add some utility and add to the trust in organisations and products. Transparency and trust are essential benefits of regulation as well as self-imposed processes aligned to ethical principles and guidelines.

The European Commission has meanwhile taken a further step just days before the cutoff point for this manuscript, in publishing a document going in the direction proposed here, i.e., rather than just asking important questions and stating requirements, this document is putting forward first recommendations for actions conducive to the responsible design of trustworthy AI systems [50].

## Data Availability

Not applicable.

## Footnotes

1 The M.O.T. test is an annual safety, roadworthiness, and exhaust check for road vehicles in the UK.
